# Solid-State Synthesis of Water-Soluble Chitosan-g-Hydroxyethyl Cellulose Copolymers

**DOI:** 10.3390/polym12030611

**Published:** 2020-03-07

**Authors:** Tatiana S. Demina, Aisylu V. Birdibekova, Eugenia A. Svidchenko, Pavel L. Ivanov, Anastasia S. Kuryanova, Tikhon S. Kurkin, Zulfar I. Khaibullin, Galina P. Goncharuk, Tatiana M. Zharikova, Sankarprasad Bhuniya, Christian Grandfils, Peter S. Timashev, Tatiana A. Akopova

**Affiliations:** 1 Enikolopov Institute of Synthetic Polymeric Materials, Russian Academy of Sciences, 70 Profsouznaya str., Moscow 117393, Russia; aisjlu14@mail.ru (A.V.B.); evgensv@yandex.ru (E.A.S.); ivanovpl@inbox.ru (P.L.I.); t.kurkin@gmail.com (T.S.K.); khaibullin.zulfar@yandex.ru (Z.I.K.); duna2011@yandex.ru (G.P.G.); akopova@ispm.ru (T.A.A.); 2 Institute for Regenerative Medicine, Sechenov First Moscow State Medical University, Sechenov University, 8-2 Trubetskaya str., Moscow 119991, Russia; stassy2202@gmail.com (A.S.K.); zharikova.tm@gmail.com (T.M.Z.); timashev.peter@gmail.com (P.S.T.); 3 Semenov Institute of Chemical Physics, Russian Academy of Sciences, 4 Kosygina St., Moscow 119991, Russia; 4 Amrita Centre for Industrial Research and Innovation, Amrita School of Engineering, Amrita Vishwa Vidyapeetham, Coimbatore 64112, India; b_sankarprasad@cb.amrita.edu; 5 Department of Chemical Engineering and Materials Science, Amrita School of Engineering, Amrita Vishwa Vidyapeetham, Coimbatore 641112, India; 6 Interfaculty Research Centre on Biomaterials (CEIB), University of Liège, Chemistry Institute, B6C, B-4000 Liege (Sart-Tilman), Belgium; c.grandfils@uliege.be; 7 Institute of Photonic Technologies, Research center “Crystallography and Photonics”, Russian Academy of Sciences, 2 Pionerskaya str., Troitsk, Moscow 142190, Russia

**Keywords:** chitosan, hydroxyethyl cellulose, solid-state co-extrusion, FTIR, elemental analysis, rheological properties, mechanical characteristics

## Abstract

Graft copolymers of chitosan with cellulose ether have been obtained by the solid-state reactive mixing of chitin, sodium hydroxide and hydroxyethyl cellulose under shear deformation in a pilot twin-screw extruder. The structure and composition of the products were determined by elemental analysis and IR spectroscopy. The physicochemical properties of aqueous solutions of copolymers were studied as a function of the composition, and were correlated to the mechanical characteristics of the resulting films to assess the performance of new copolymers as coating materials, non-woven fibrous materials or emulsifiers for interface stabilization during the microparticle fabrication process.

## 1. Introduction

The polysaccharide chitosan, consisting of glucosamine and N-acetylglucosamine units, is a non-toxic biocompatible polymer with unique complexing properties and solubility in dilute aqueous organic and mineral acids. The modification of the chitosan chemical structure to improve its solubility in neutral aqueous media can significantly expand the areas of its biomedical use, such as when working with pH-sensitive drugs like as those used in oncology. Following this purpose, a number of chitosan derivatives—or mixtures with additives made of synthetic polymers, including polyelectrolyte complexes—have been proposed to improve the physicochemical and mechanical properties of materials based on this polysaccharide [1,2,3]. The chemical modification of chitosan has been mostly focused on its amino groups, which often leads to a suppression of its complexing properties. A particular alternative relies on the synthesis of copolymers of chitosan with nonionic water-soluble polymers. Several authors have reported the synthesis of copolymers of chitosan with polyacrylamide [4,5], polyethylene glycol [6,7] and polyvinylpyrrolidone [8], all of which are soluble in water at neutral or close to pH values. Moreover, the copolymerization of chitosan with nonionic water-soluble fragments changes its solubility, and could allow using chitosan with a low degree of deacetylation (DD), which normally decreases its solubility according to a classical dissolution procedure at acidic pH via the protonation of amino groups, but also decreases its toxicity [9]. The bioactivity of chitosan depends greatly on its DD in terms of binding/releasing efficiency (including drugs, DNA, etc.) [10,11], cellular uptake [9] and the biodegradation rate [12]. Chitosan amino groups are widely used as a conjugating site in drug delivery systems, while the decrease of the polysaccharide Mw to several kDa is required to preserve its solubility in an aqueous medium [13]. Therefore, a possibility to play on chitosan DD without compromising its solubility, as well as the possibility to work at a neutral pH, is particularly attractive for the application of synthesized copolymers as drug delivery vehicles, both in the form of macromolecular platforms and solid micro-/nanoparticles or capsules. Moreover, water-soluble chitosan copolymers have great potential for the fabrication of cell carriers/capsules and bioinks [14,15]. However, the preparation of copolymers by conventional liquid-phase methods is associated with well-known disadvantages, such as multi-stage, long process time, the use of toxic catalysts and low yield.

This work is aimed to prepare graft copolymers of chitosan and hydroxyethyl cellulose (HEC) under the simultaneous action of pressure and shear deformation onto solid polymer mixtures. As it validates by a number of experiments, the mechanical activation of polymers and their intensive intermixing enhances the activation of functional groups of polymers, which would be inactive under ordinary conditions, and often inaccessible in liquid media [16,17,18]. We could expect to generate chitosan-g-hydroxyethyl cellulose copolymers using this methodology after fine-tuning the terms of mechanochemical synthesis.

For the chemical modification of polymers in the solid state (the most concentrated systems), twin-screw extruders containing strength elements in stacked screws are more suitable for the efficient compression and deformation of reactive mixtures. In recent times, such equipment was mainly used for processing pastes in the preparation of additives. However, the possibilities of the solvent-free extrusion method for conducting solid-state organic reactions has gained more attention in more recent days [19,20,21,22]. In this way, extrusion processes performing the derivatizing and grafting of polysaccharides are very interesting, as they require the activation of their functional groups stacked within the native highly organized supramolecular structures. Due to high cohesion energy between macromolecular chains, polysaccharides have a poor solubility, and cannot resist melting without decomposition. A feature of our approach is to use chitosan straight away after its preparation from chitin by solid-state alkaline deacetylation, under the same conditions as it is chosen for its reactive mixing with HEC.

The aim of this work is to synthetize water-soluble chitosan-g-hydroxyethylcellulose copolymers through a mechanochemical approach, and evaluate their applicability for the fabrication of various materials tailored for biomedical applications.

## 2. Materials and Methods

Chitosan with a molecular weight (*M*_w_) of 80 kDa and a degree of deacetylation of 0.85 was synthesized via mechanochemical approach, as described earlier [23]. Commercially available hydroxyethyl cellulose (HEC) of L250 Natrosol type (Ashland, Covington, KY, USA) with *M*_w_ of 90 kDa was kindly received from the company as a sample for research, and was used without additional treatment.

The synthesis of the copolymer samples (marked as ChsHEC) was carried out in a pilot industrial twin-screw extruder of the brand “Berstorff”, with a screw diameter of 40 mm at a temperature of 160 °C to obtain chitosan, and at 50 °C the blending of the polymers. We used a three-fold molar excess of NaOH per 1 unit of chitin, which is significantly low in contrast to the known suspension methods (at least 10 moles of NaOH). For the samples of ChsHEC-1 and ChsHEC-2 synthesized with chitosan (without prior purification) as a semi-product, the total amount of NaOH in the reactive mixture of polysaccharides was 8 and 18 wt %, respectively. The ChsHEC-3 was co-extruded using pure chitosan washed primarily by water and freeze-drying. Samples synthesized in the presence of alkali were subjected to dialysis against water using membranes (ZelluTrans/Roth) with a molecular weight cut-off of 3500 until neutral pH, and then freeze-dried.

The fractional analysis of all samples was carried out as follows: a sample (~3 g) was dissolved by stirring in 100 mL of Type II water for 2 h. These water-soluble fractions were separated by centrifugation, freeze-dried and weighted. The insoluble-in-water fractions were repeatedly washed with Type II water, freeze-dried and weighted, and then dissolved in 100 mL of 2% acetic acid at RT for 2 h. Insoluble-in-acidic-medium fractions were separated by centrifugation, washed with Type II water, freeze-dried and weighted, and soluble-in-acetic-acid fractions were precipitated with 1 M NaOH, collected by centrifugation, washed with Type II water and freeze-dried.

The content of carbon, nitrogen and hydrogen was revealed using a FLASH-2000 Organic Elemental Analyzer (Thermo Fisher Scientific, Waltham, MA, USA). The glucosamine content was calculated from the EA data using a C/N ratio for both pure and modified chitosan.

FTIR-spectroscopy of water-soluble (at neutral pH) fractions in the form of film samples were carried out in an ATR mode using a Nicolet iS50 FTIR Spectrometer (Thermo Fisher Scientific, Waltham, MA, USA) with an in-built diamond crystal ATR accessory. Prior to measurements, diamond crystal was cleaned using CCl_4_ to remove surface residuals. The spectra were normalized using an intensity of C–O stretching vibrations of pyranose cycle band (1080 cm^−1^) as an internal standard, with the aim of Bruker Opus (version 6.1, Bruker, Billerica, MA, USA) [24].

The viscosity (η) of 1 and 10 wt % solutions of initial polymers and water-/acid-soluble fractions of the copolymers was measured using an electromagnetically spinning viscometer EMS-1000 (Kyoto Electronics Manufacturing, Kyoto, Japan) at 25 °C, after a rest time of 3–5 min, and applying a shear rate of 400 s^−1^. The electrical conductivity of polymeric solutions in Type II water was also evaluated with an aim of Expert-002 conductometer (Volta, Saint Petersburg, Russia). Surface tension was determined on a Du-Nui tensometer by a platinum ring detachment.

Dynamic laser scattering (DLS) of 1 wt % water solutions of non-modified HEC and water-soluble fractions of ChsHEC samples, as well as a solution of initial chitosan in 0.1 M HCl, was carried out using a Zetatrac particle size analyzer (Microtrac, Inc., Montgomeryville, PA, USA).

UV/Vis-spectrophotometry of 1% solutions of parent polymers and synthetized ChsHEC samples was carried out in quartz cells with an optical path length of 1 cm, using a Shimadzu UV 2501 PC spectrophotometer.

Films from HEC, chitosan, their blends at various ratio and water-/acid soluble fractions of the copolymers were fabricated through the casting of 2 wt % respective polymeric solutions either in Type II water or 2% acetic acid on a polystyrene Petri dish, and drying in a dust-free chamber at RT. The blend solutions were prepared by mixing (using a four-bladed propeller stirrer at 500 rpm for 10 min) calculated amounts of initial component solutions, i.e., HEC in water and chitosan in 2% CH_3_COOH, to achieve a desired component ratio and targeted final solution concentration, i.e., 2 wt %. The thickness of the films was 50–100 μm. Before mechanical testing, the films were kept in a desiccator at a constant humidity of 81% above the (NH_4_)_2_SO_4_ saturated solution for a week. Mechanical studies of the film samples were carried out using an AGS-H universal testing machine (Shimadzu, Kyoto, Japan) at a speed of 1 mm/min. Mechanical characteristics of the films, namely tensile strength, elastic modulus and elongation at break, were calculated as the average value of five measurements of a film sample, taking into account its thickness using the WinAGS Lite Cycle instrument software (version 14.4, Shimadzu, Kyoto, Japan).

Polylactide microparticles stabilized with water-soluble fractions of the ChsHEC copolymers were fabricated according to previously described procedure with minor modifications [19]. A 5 wt % solution of poly(L,L-lactide) (Natureworks 4043D, Minnetonka, MN, USA) in a CH_2_Cl_2_/acetone (9/1 *v/v*) mixture was used as an oil phase, and emulsified within the aqueous phase, consisting of 1 wt % solutions of parent polymers (non-modified chitosan was used in a form of the solution in 2% AcOH) or water-soluble fractions of ChsHEC samples. The oil/water phase ratio was 1/9 *v/v*. The samples were stirred at 700 rpm using a four-bladed propeller stirrer at a controlled temperature until solvents from the oil phase were evaporated. Formed polylactide microparticles were simultaneously washed with Type II water and sieved, and then freeze-dried. The total yield of the fabricated microparticles was calculated as the ratio between weights of recovered microparticles to the total amount of polylactide dissolved in the oil phase for each system.

Scanning electron microscopy (SEM) of the films and microparticles was carried out using a PhenomProX (PhenomWorld, Eindhoven, Netherlands), operated at 10–15 kV.

## 3. Results

### 3.1. Chemical Analysys

#### 3.1.1. Fractional Analysis

Pre-mixed batches (400 g) of HEC with chitosan were processed in an extruder at various ratios of components. Solid mixtures were gradually fed into the extruder in a predetermined range of heating zones. All co-extruded samples were subjected to fractional analysis by their sequential dissolution in water at neutral pH (feature of HEC), an acidic aqueous solution (feature of chitosan) and insoluble-in-aqueous media fragments, which could be attributed in the presence of chitosan macromolecules with a high content of remaining N-acetyl glucosamine units or cross-linked products of reactions between HEC and chitosan. The data of fractional analysis given in Table 1 shows that the amount of insoluble-in-aqueous media fraction of ChsHEC-1 and 2 corresponds to content within the chitosan taken for the synthesis, while ChsHEC-3 showed a prominent increase of this fraction, which partially consists of a gel-like substance, indicating a formation of relatively cross-linked material at chosen conditions of synthesis. This is probably due to the recombination of macro radicals formed during the mechanically induced destruction of polymer chains [25,26,27,28]. The presence of alkali in the reactive mixture, as a mechanically softer material, obviously hinders these processes, reducing the mechanical action onto the polymers.

#### 3.1.2. Elemental Analysis

A qualitative and quantitate analysis of water-soluble at neutral pH copolymer fractions was carried out using ninhydrin assay and elemental analysis (EA), in order to reveal the presence of chitosan fragments. While the ninhydrin assay confirmed the presence of amino groups within all studied fractions, the data of elemental analysis allowed estimating the actual content of chitosan units drawn in water. EA data and the calculated content of chitosan within water-soluble fractions are given in Table 2. The calculations showed that in the ChsHEC-2 fraction most enriched in chitosan, its solubility in neutral water is achieved with a relatively low molar content of grafted HEC units.

#### 3.1.3. FTIR-Spectroscopy

**Figure 1:**
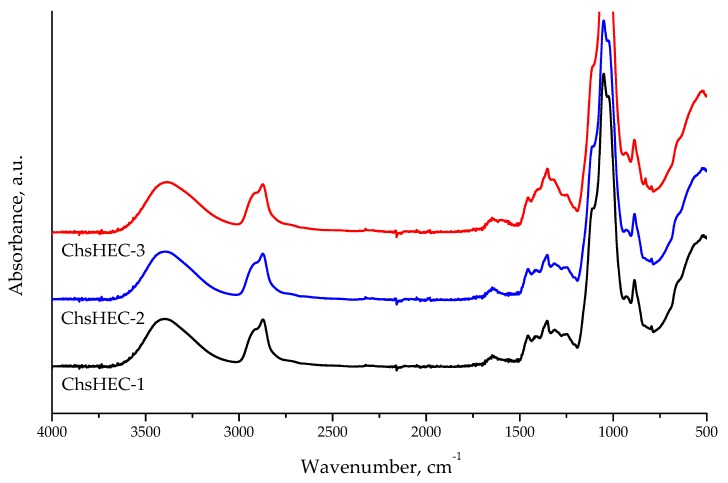
IR-spectra of water-soluble (at neutral pH) fractions of the ChsHEC samples.

FTIR-spectra of water-soluble at neutral pH fractions of the ChsHEC samples are shown in Figure 1. The spectra of all samples contain an overlapping band assigned to chitosan-NH_2_ bending at 1597 cm^−1^ and Amid-I vibrations of the residual acetamide groups of chitin at 1650 cm^−1^.

#### 3.1.4. UV/Vis-Spectrophotometry

**Figure 2:**
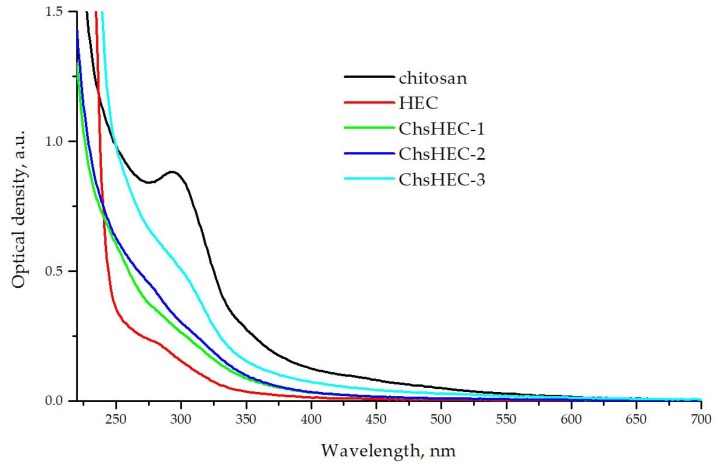
Electron absorption spectra of 1% solutions of initial chitosan in 0.1 M HCl, aqueous solution of initial HEC and water-soluble fractions of ChsHEC samples.

The absorption spectra of 1% solutions of the starting materials (chitosan in a 0.1 M solution of HCl and HEC in water) and the co-extruded samples (ChsHEC-1, 2, 3 in water) were analyzed after subtracting the contribution of the solvent (Figure 2). All the solutions were characterized by Rayleigh scattering, with an equation of dependence of the scattering coefficient on the wavelength close to four. This observation is indicative that the particle size of all these copolymer solutions is many times smaller than the wavelength of the incident light.

#### 3.1.5. Dynamic Light Scattering

DLS measurements of 1 wt % aqueous solutions of initial HEC and water-soluble fractions of ChsHEC samples showed a presence of nano-sized particles (macromolecular aggregates) with a mean size of 10–12 nm. However, in contrast to non-modified HEC, the copolymer samples showed a minor amount of macromolecular associates with a size of approximately 530 nm, which could be attributed to chitosan fragments since a solution of non-modified chitosan in 2% acetic acid showed a presence of aggregates with the same size.

#### 3.1.6. ChsHEC Solution Characteristics

The main characteristics of aqueous solutions of ChsHECs, such as viscosity, conductivity and surface tension, are presented in Table 3. 10% polymer solutions were prepared in order to evaluate their suitability for electrospinning techniques.

### 3.2. Materials Based on the Copolymers

#### 3.2.1. Films

**Figure 3:**
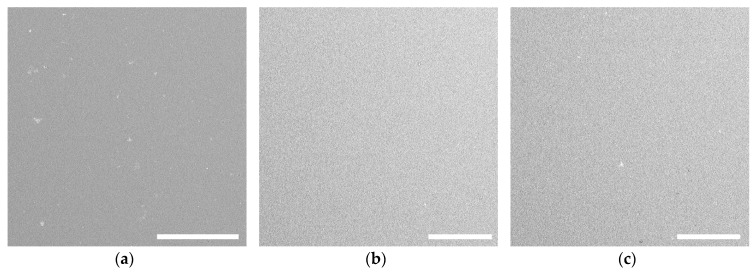
SEM images of the films cast from 2 wt % solutions of (**a**) HEC, (**b**) chitosan and (**c**) water-soluble fraction of ChsHEC-2. Scale bar is 100 μm.

The parent polymers and the ChsHEC samples disclosed good film-forming ability. As could be seen in Figure 3, all of the films possess homogeneous surface morphology.

Table 4 summarized the mechanical tests of the films made of homopolymers, their physical blends and the copolymers. The results demonstrate that the mechanical properties of the copolymer differ significantly from the homopolymer films, so even a small content of chitosan in copolymer films (ChsHEC-1 sample) leads to an increase in both the tensile strength and plasticity of the films from the HEC (24 vs. 13 MPa and 52 vs. 33%, respectively). The same observation was made in the case of chitosan homopolymer films, in which tensile strength was increased by 23% due to the graft of no more than 2 wt % of HEC fragments.

#### 3.2.2. Polylactide Microparticles Stabilized with the Copolymers.

**Figure 4:**
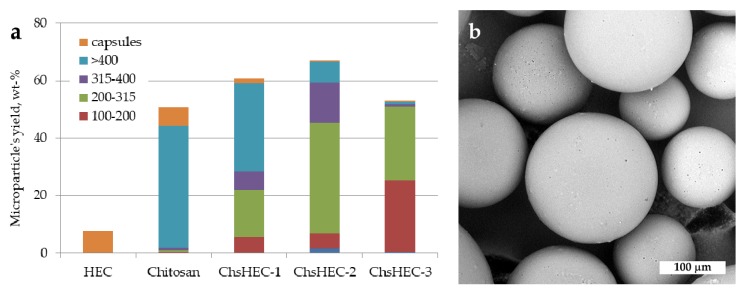
(**a**) Histogram of microparticle’s total yield and size distribution as a function of polymer used as an emulsifier in the aqueous phase; (**b**) micrograph of polylactide microparticle stabilized with 1 wt % aqueous solution of ChsHEC-2.

The effectiveness of the water-soluble fractions of the ChsHEC samples to stabilize oil/water interface for the fabrication of polylactide microparticles via the oil/water solvent evaporation technique was evaluated. As could be seen in Figure 4a, the water-soluble fractions of the co-extruded samples allowed ensuring a higher total yield of the microparticles in comparison with the homopolymers. The enhanced emulsifying ability of the prepared samples led to the production of smaller microparticles, i.e., the stabilization of a larger interface area. The resulting microparticles, irrespective of the ChsHEC copolymer used as an emulsifier, have a spherical shape and a homogeneous surface morphology.

## 4. Discussion

**Figure 5:**
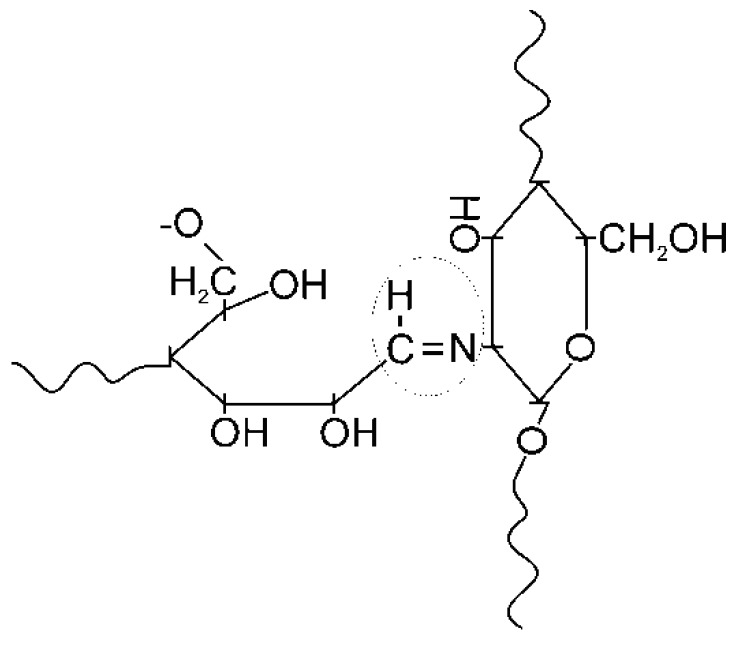
The formation of aldimine bonds during the interaction of the aldehyde end groups of HEC and amino groups of chitosan.

The copolymerization of polysaccharides is a very tricky task, due to the high molecular weight of co-reagents and the complexity of their chemical structure, which is highly organized at the supramolecular level. Solid-state reactive extrusion offers unique advantages for carrying out the chemical modification of chitosan, combining two main effects: the removal of diffusion restrictions due to the plastic deformation of multicomponent solid mixtures and the formation of solid solutions at the molecular-segmental level during the simultaneous deformation of macromolecular and low-molecular-weight substances [23]. This specific treatment can promote the reaction of the aldehyde end groups of HEC with the amino groups of chitosan with the formation of aldimine bonds (a polymer analog of Schiff bases). The proposed scheme of interaction is presented in Figure 5. This reaction accelerates in the presence of alkali, and is widely used for the selective N-alkylation of chitosan and its crosslinking by dialdehydes [29].

The covalent binding of chitosan with HEC in the course of mechanochemical treatment was confirmed by a study of the composition and structure of water-soluble fractions of the samples after co-extrusion (42–77 wt % yield). The results showed the presence in them of up to 18 wt % of chitosan, which is usually insoluble in water at neutral pH values (Table 1 and Table 2).

Figure 1 presents the overview IR-spectra of water-soluble fractions of the samples. The high-frequency part (3700–2500 cm^−1^) of the spectra contains a strong broad band of the stretching vibrations of O–H groups in the structure of both chitosan and HEC macromolecules, which overlaps with N–H stretching vibrations. The doublet of C–H stretching vibrations can be clearly seen with a maximum of 2870 cm^−1^, and its high-frequency shoulder at approximately 2990 cm^−1^. The spectra of all soluble at neutral pH ChsHEC samples contain an overlapping band at a maximum of 1600 cm^−1^, which can be assigned to chitosan -NH_2_ bending at 1597 cm^−1^ and Amid-I vibrations of the residual acetamide groups of chitin at 1650 cm^−1^ [30,31,32]. The participation of NH_2_ groups in reactions could nonetheless be seen due to the broadening and low-frequency shift towards the Amide-II band (down to 1550 cm^−1^). The weak band at approximately 920 cm^−1^, according to the literature [32], could be attributed to =C–H deformation vibrations. This coincides well with our conclusion about the character of HEC interaction with chitosan. The intensity of the NH_2_ groups band, as well as EA data (see Table 1), confirm a relatively small amount of chitosan units in the copolymer samples.

The presence of chitosan fragments within water-soluble fractions of copolymers was also confirmed by UV/Vis-spectrometry. As could be seen in Figure 2, the UV/visible absorption spectrum of the initial chitosan highlights a band with a maximum of 294 nm, as well as a band of lower intensity with a maximum of 350 nm (appears as a shoulder) and an intense short-wave band with a maximum lying in the region of vacuum UV. The absorption spectrum of the initial HEC shows a broad absorption band with a maximum of 280 nm, which is about four times weaker than the absorption band of chitosan in the close region (294 nm), and an intense short-wave band with a maximum lying in the region of vacuum UV. The addition of chitosan units to the HEC is accompanied by an increase in the intensity of the absorption band, with a maximum of 280 nm.

The formation of water-soluble chitosan copolymers opens a number of doors to the application of the solutions itself, or the formation is based on materials loaded by sensitive components. Thus, a considerable part of the work is related to an investigation of the solutions and the possibility to form several materials. The viscosity of the ChsHEC systems was low enough, allowing us to use its solutions for drug delivery. It was revealed that all samples under investigation form homogeneous flowing solutions with a polymer concentration of up to 10%, the appearance and viscosity of which does not change during storage for one week. The viscosity of the all co-extruded sample solutions was lower than that of parent polymers, supporting the assumption of mechanically-induced and/or oxidative destruction of polymer chains during mechanical treatment (Table 3). From this viewpoint, it seems to be logical that the most significant decrease in the viscosity of fraction solution both in neutral aqueous and acidic media was observed for sample 2, prepared at a sufficiently high alkali content in the initial mixture. It can be assumed that the composition of this copolymer contains the shortest chains of the parent polymers. This prototype also explains its solubility in neutral water at a relatively low molar content of grafted HEC fragments (Table 2). It affected the film-forming ability of this sample, and did not allow us to evaluate the mechanical characteristics of the material made of the chitosan-enriched fraction. However, the blending of HEC with chitosan generally results in the enhancement of mechanical properties of the composite and copolymer films. So, even a small content of chitosan in copolymer films (ChsHEC-1 sample) leads to an increase in both the tensile strength and plasticity of the films from the HEC (24 vs. 13 MPa and 52 vs. 33%, respectively). The same observation was made in the case of chitosan homopolymer films, in which tensile strength increased by 23% due to the graft of no more than 2 wt % of HEC fragments.

Taking into account a potential use of the fabricated copolymers for the development of new non-woven chitosan-based mats via an electrospinning technique, other specific solution characteristics of water-soluble fractions of ChsHEC copolymers, such as conductivity and surface tension, were evaluated. The electrospinning of chitosan-based materials is of great interest due to the high perspectives of application of non-woven mats in tissue engineering [33,34,35]. Since the electrospinning of non-modified chitosan is hindered by the need to use concentrated acids (up to 90%) as a solvent [36] or additional components of a casting solution [37], the possibility of using copolymer solutions in water could be a significant benefit. The conductivities of 1% water solutions of the copolymers are similar to that of HEC, which has a good agreement with a relatively small amount of chitosan units in the water-soluble copolymer fractions confirmed by FTIR spectra (Figure 1) and EA data (Table 2). In the meantime, 10% copolymers solutions, which are more suitable for the electrospinning process, show the values of viscosity and conductivity, which are ideal for this purpose. Surface tensions of 1% and 10% water solutions of the copolymer fractions were lower than that of initial components, but higher than those required for a stable electrospinning process. A detailed study of the suitability of the obtained copolymers to the electrospinning of chitosan-containing non-woven mats will be the subject of our further research.

Other forms of scaffolds for tissue engineering (such as hydrogels) are of great importance and open for polysaccharide-based materials. Among a variety of polymers used for the fabrication of hydrogels for neuroregeneration, chitosan and cellulose derivatives are widely recognized [38,39].

A combination of fragments having a different solubility in aqueous media at neutral pH within one macromolecule is also fruitful, in terms of the further application of these copolymers as an emulsifier. The synthesized copolymers were successfully used as interface stabilizers during a fabrication of polylactide microparticles via an oil/water solvent evaporation technique. In contrast to parent materials, the application of the copolymers led to a formation of spherical microparticles with a high total yield (up to 67 wt %), which was comparable to those obtained with an aim of traditional emulsifiers (polyvinyl alcohol) or other chitosan-based copolymers [14]. Water-soluble chitosan-based emulsifiers could be especially useful for the fabrication of drug-loaded or composite microparticles containing pH-sensitive components. For example, fabricated microparticles could be used as biodegradable multifunctional drug-loaded cell microcarriers. Such a type of advanced carrier contains a bioactive component within a polymeric core, and a surface layer supporting the adhesion and growth of anchorage-depended cells [40]. Since biodegradable particles are promising materials either in the form of nano/micro-particles as a drug/cell carrier, or as a starting material for the fabrication of 3D structures, developing new types of microparticles is of great importance [41,42].

Therefore, we conclude that the solid-state reactive mixing of chitin, sodium hydroxide and hydroxyethyl cellulose under shear deformation in a pilot twin-screw extruder led to the formation of water-soluble graft copolymers of chitosan with cellulose ether, with a chitosan content of up to 18 wt %. The obtained copolymers are promising for creating physiologically suitable conditions for new biomedical materials, primarily in the form of hydrogels, as well as in the form of fibers and films.

## Figures and Tables

**Table 1:** Solubility of the samples in various aqueous media.

Sample Code	Initial Polymers Composition, wt %	Content of Fraction, wt %		
		Soluble in Water	Soluble in 2% CH_3_COOH	Insoluble in 2% CH_3_COOH ^3^
ChsHEC-1 ^1^	Chitosan-20 HEC-80	77.6	19.8	2.6
ChsHEC-2 ^2^	Chitosan-50 HEC-50	42.7	49.8	7.5
ChsHEC-3	Chitosan-50 HEC-50	47.3	30.6	22.1

^1^ the mixture was treated in the presence of 8 wt % of NaOH; ^2^ the mixture was treated in the presence of 18 wt % of NaOH; ^3^ initial chitosan contains 14.7 wt % of insoluble in 2% CH_3_COOH fraction.

**Table 2:** Elemental analysis data of water-soluble at neutral pH fractions and the calculated chitosan content.

Sample Code	Atomic Concentration, wt %			Chitosan Content, wt %	Chitosan/HEC Unit Ratio
	C	H	N		
ChsHEC-1	47.50	7.42	0.11	1.3	1/76
ChsHEC-2	47.75	7.33	1.52	18	1/4
ChsHEC-3	45.18	7.04	1.03	12	1/7

**Table 3:** Characteristics of the aqueous solutions of initial polymers and ChsHEC samples.

Sample Code	η, mPa·s	Conductivity, mS·cm^−1^	σ, mN/m
HEC ^4^	4 ± 0.02	0.3 ± 0.01	66 ± 0.2
Chitosan ^4,6^	6.7 ± 0.05	8.3 ± 0.2	63 ± 0.3
ChsHEC-1 ^4^	3.5 ± 0.01	0.3 ± 0.01	62 ± 0.1
ChsHEC-2 ^4^	2.7 ± 0.02	0.4 ± 0.01	60 ± 0.1
ChsHEC-3 ^4^	3.3 ± 0.02	0.3 ± 0.02	61 ± 0.2
ChsHEC-1 ^5^	434 ± 1	0.8 ± 0.01	60 ± 0.1
ChsHEC-2 ^5^	176 ± 10	2.4 ± 0.1	56 ± 0.1
ChsHEC-3 ^5^	397 ± 2	0.8 ± 0.03	60 ± 0.1

^4^ 1% polymer solution; ^5^ 10% polymer solution; ^6^ 1% solution in 2% AcOH.

**Table 4:** Characteristics of composite chitosan/HEC films and the films cast from various fractions of the ChsHEC copolymers.

Sample Code	Chitosan Content, wt %	σ, MPa	ε, %	E, MPa
HEC	0	13 ± 1	33 ± 2	44 ± 4
Chitosan	100	47 ± 5	20 ± 4	1900 ± 200
Chitosan/HEC	20	48 ± 5	40 ± 6	720 ± 100
Chitosan/HEC	50	39 ± 5	31 ± 6	1100 ± 130
Chitosan/HEC	80	50 ± 2	33 ± 4	1980 ± 130
ChsHEC-1 ^7^	1.3	24 ± 3	52 ± 3	480 ± 40
ChsHEC-2 ^7^	18	32 ± 4	25 ± 1	1340 ± 130
ChsHEC-3 ^7^	12	21 ± 1	39 ± 2	560 ± 50
ChsHEC-1 ^8^	98.7	58 ± 2	23 ± 4	2900 ± 100
ChsHEC-3 ^8^	88	66 ± 3	13 ± 2	2100 ± 400

^7^ water-soluble fraction; ^8^ acid-soluble fraction.

## Abbreviations

HEC	Hydroxyethyl cellulose
EA	Elemental analysis
UV	Ultra violet region
DD	Degree of deacetylation
Mw	Molecular weight
DLS	Dynamic light scattering
